# Prevalence and predictors of anemia among pregnant women in Ethiopia: Systematic review and meta-analysis

**DOI:** 10.1371/journal.pone.0267005

**Published:** 2022-07-27

**Authors:** Teshome Gensa Geta, Samson Gebremedhin, Akinyinka O. Omigbodun

**Affiliations:** 1 Department of Biomedical Science, College of Medicine and Health Science, Wolkite University, Wolkite, Ethiopia; 2 Pan African University, Life and Earth Science Institutes (Including Health and Agriculture) Ibadan, Nigeria; 3 School of Public Health, College of Health Science and Medicine, Addis Ababa University, Addis Ababa, Ethiopia; 4 Department of Obstetrics & Gynaecology, College of Medicine, University of Ibadan, Ibadan, Nigeria; Georgetown University, UNITED STATES

## Abstract

**Background:**

In Ethiopia limited information is available regarding the prevalence and predictors of anemia in pregnancy. This systematic review and meta-analysis estimated the pooled prevalence of anemia among pregnant women in Ethiopia and also identified its predictors.

**Materials and methods:**

The published primary studies were searched in the following electronic databases; PubMed/Medline, Google scholars, AJOL, and EMBASE. All primary studies published from 01/01/2010 to 30/05/2020 and written in English language were included without restriction on study setting and design. Critical appraisal of all available articles was done and extracted data was analyzed using STATA software version 14. The pooled prevalence of anemia was presented using a forest plot. The I^2^ statistical test for heterogeneity, and the Egger’s and Begg’s tests for publication bias were used. The relative risk was used to assess the association of predictor variables with anemia.

**Result:**

After screening 274 articles, sixty studies were included in the analysis. The pooled prevalence of anemia among pregnant women was 26.4(95% CI: 23.1, 29.6). Sub-group analysis showed higher pooled prevalence from community-based studies than institutional-based studies. Factors that were protective against maternal anemia included urban residence, formal education and smaller family size. Short birth interval and not having antenatal care (ANC) are associated with a higher risk of maternal anemia. Women with low dietary diversity [RR: 2.61(95% CI, 1.85, 3.68)], mid-upper arm circumference (MUAC) less than 23 cm [RR: 2.35(95% CI, 1.53, 3.68)] and those not taking iron-folic acid [RR: 1.53(95% CI: 1.30, 1.81)] also had a higher risk of anemia.

**Conclusion:**

Almost one in four pregnant women in Ethiopia had anemia. Being literate, living in urban areas with small family size and adequate birth spacing, as well as good dietary diversity are associated with a lower risk of anemia in pregnancy.

**Registration number:**

(ID: CRD42020211054).

## Introduction

Anemia is defined as a reduced number of circulating red blood cells (RBC) or a condition in which the number of RBC or its oxygen-carrying capacity is insufficient to meet physiological needs [1]. Hemoglobin concentration is the most common hematological assessment method used to define anemia. The level of hemoglobin concentration to fulfill the body demand varies with a person’s age, gender, altitude, pregnancy, and other health-related behaviors. The hemoglobin threshold to define anemia therefore varies in different population. The World Health Organization (WHO) defines anemia for pregnant women as a hemoglobin concentration less than 11g/dl at sea level [2].

Anemia is a global public health problem affecting all age groups in developing and developed countries [3]. Globally, a total of 1.93 billion people are living with anemia [4]. Among this population, women of reproductive age group, particularly pregnant women, are among the most vulnerable group. According to a WHO report, 29% of non-pregnant women and 38% of pregnant women aged 15–49 years were anemic in 2011 [5]. The highest prevalence of anemia among pregnant women was from sub-Saharan Africa, 38.9% to 48.7% [6]. Ethiopia is one of the countries that share this burden. The Ethiopian Demographic Health Survey 2016 (EDHS) showed the overall prevalence of anemia among pregnant women to be 41%, of which 20% were moderately anemic, 18%, mildly anemic and 3%, severely anemic [7].

Anemia during pregnancy is an important predictor of poor pregnancy outcomes such as low birth weight (LBW), prematurity, stillbirth, and intrauterine growth restriction [8, 9]. It is also associated with small for gestational age, low Apgar score, perinatal and neonatal death [10]. Women with anemia have a high risk of maternal morbidities such as abortions, antepartum hemorrhage, postpartum hemorrhage, preeclampsia, and prolonged labor [11, 12].

The causes of anemia in pregnancy are multi-factorial. Increased nutritional demand and unmet need for micronutrients are associated with anemia during pregnancy. Intestinal helminthic infections, malaria, and chronic illnesses are comorbidities that increase the risk of maternal anemia [13]. Other factors that contribute to the high burden of anemia among pregnant women in developing countries include low socioeconomic status, rural residence, decreased birth interval, late ante-natal clinic initiation, grand multiparty, and during the third gestational trimester [14, 15].

The dietary pattern and nutritional status of women during and before pregnancy is an important predictor of anemia among pregnant women. Reduced meal frequency and low dietary diversity among pregnant women were associated with anemia [16]. Non-compliance and insufficient uptake of iron supplementation are also contributing factors to it [17]. Moreover, the nutritional status of women; assessed by mid-upper arm circumference (MUAC) and body mass index (BMI), were associated with anemia [14]. Maternal malnutrition is one of the important problems in Ethiopia where 27% of women were undernourished [18]. Hence, the nutritional status of women is one of the prevalent factors that increase the risk for maternal anemia.

Maternal anemia is multifactorial and despite many interventions targeted to reduce the burden of anemia among pregnant women, it remains a public health issue in Ethiopia [16]. This may be due to the multiplicity and complexity of contributing factors. Despite many studies on the subject of anemia, there was a scarcity of adequate and organized information on predictors of anemia in pregnancy. So, this systematic review and meta-analysis was conducted to estimate the pooled prevalence of anemia during pregnancy at the national and regional levels. Moreover, it also identified predictors of anemia among pregnant women by reviewing primary studies readily available in different databases. This information will help policymakers and health professionals to design effective and efficient strategies to mitigate the problem.

## Materials and methods

### Information sources and search strategy

The systematic review and meta-analysis was done by using published studies. The articles were searched in the following electronic databases; PubMed/Medline, Google scholars, AJOL, and EMBASE.

The key terms and search strategies used for PubMed database were (((associated factors [Title/Abstract] OR risk factors [Title/Abstract]) OR ((prevalence [Title/Abstract] OR magnitude [Title/Abstract]) OR "burden"[Title/Abstract])) OR prevalence[MeSH Terms]) AND Anemia [Title/Abstract]) OR "Anemia" [MeSH Terms])) AND pregnant women [Title/Abstract] AND (Ethiopia [Title/Abstract] OR "Ethiopia"[MeSH Terms]). The key terms and titles were also used to search articles from the above-mentioned relevant databases other than PubMed.

Standard guidelines were used to organize and report the study. The Preferred Reporting Items for Systematic Reviews and Meta-Analyses (PRISMA) guidelines [19] and meta-analysis of observational studies in Epidemiology (MOOSE) guidelines [20] were used to report the finding. The protocol for the systematic review and meta-analysis was registered in the International Prospective Register of Systematic Reviews (PROSPERO) (ID: CRD42020211054).

### Eligibility criteria

All primary studies that reported the prevalence and predictors of anemia among pregnant women in different regions of Ethiopia and published from 01/01/2010 to 30/05/2020, were included. All accessible full-text articles written in the English language and conducted by cross sectional study, case-control, retrospective and prospective study designs were included without restriction on study setting. The secondary reports and un-published articles were excluded from the study.

### Study selection

After collecting all the available published articles, the duplicates were removed using *Endnote*. Then the title and abstract of all the studies were reviewed. The full-text review has been done for relevant articles left after the title and abstract review. Finally, articles with no variable of interest and low quality were excluded.

### Variables

#### Outcome of interest

The primary outcome of this systematic review and meta-analysis was the prevalence of anemia among pregnant women. Hemoglobin concentration less than 11g/dl irrespective of gestational age is considered as anemia among pregnant women.

#### Predictor variables

All the following predictor variables included in the study were reported in two or more culled papers while variables of interest in less than two papers or not reported at all were not considered.

- Socio-demographic history such as residence (rural and urban), marital status (married, non-married), education (illiterate, formal education), occupation (housewife, other jobs), and family size (less than five, greater than or equal to 5 members)
- Obstetric history such as birth interval (less than 24 months, greater than or equal to 24 months), trimester (3^rd^ trimester, 1^st^ or 2^nd^ trimester), gravidity (multigravid, primigravid), ANC follow up (yes, no), and history of excess menstrual bleeding (yes, no). Excess menstrual bleeding was defined as needing to change menstrual hygiene products every four hours and bleeding lasting longer than 7days.
- Medical history such as malaria infection (yes, no), intestinal parasitic infection (yes, no), HIV infection (yes, no), and chronic disease (yes, no).
- Nutrition status of women such as MUAC(less than 23, greater than or equal to 23), iron and folate (supplementation vs no supplimentation), dietary diversity (low, medium or high). The dietary diversity is the number of different food items or food groups consumed over a given reference period.

### Quality assessment

The quality of all the studies was assessed by critical appraisal using the Joanna Briggs Institute Meta-Analysis of Statistics Assessment and Review Instrument (JBI-MAStARI) [21]. This critical appraisal tool addresses the methodological section of the study. It includes sample representativeness, appropriate recruitment of participants, sufficient description of participants, using of standard measurement and its reliability, appropriate statistical analysis done with sufficient coverage of identified sample size, appropriate statistical analysis done, and subgroup or confounding variables identified. All the components of quality assessment were addressed in detail. Studies with a score greater than or equal to 6 out of 10 were considered as high quality and those studies with less than 6 out of 10 considered as low quality. All the available studies containing variable of interest were scored six and above.

### Data extraction process

All necessary data were extracted by using the prepared Microsoft excel data extraction format. The data extraction format includes the first author’s name, year of publication, study design, sample size, and prevalence of anemia. The format will also include expected predictors and the prevalence of anemia among women with and without exposure to those predictors. Only predictor variables were extracted from case-control studies. Two reviewers’ extracted data and any disagreement on the extracted data was resolved by rechecking the article to reach in common conscience. Inconsistent and incomplete data was removed after data extraction.

### Data analysis

Data was extracted on Microsoft excel and transported to STATA software version 14 for further analysis. The general characteristics of original articles, such as name of first author, publication year, area and setting of the conducted study, study design and sample size were presented using a table. The prevalence and its standard error of each original article were considered to calculate the pooled prevalence of anemia. The pooled prevalence was presented using a forest plot. The heterogeneity among prevalence of anemia in the studies was tested by I^2^ statistical test and a p-value less than 0.05 was used to declare it. For the test with significant heterogeneity between the studies, a random effect meta-analysis model was used. A meta-regression model was used to detect the possible sources of any heterogeneity.

The potential publication bias was assessed by using Egger’s and Begg’s correlation test at a 5% significance level. Finally, to reduce the random variation between point estimates of the primary study, subgroup analysis was done based on regions and study settings. To show the association between predictor variables and outcome variables, relative risk (RR) with a 95% confidence interval was used.

## Results

### Study selection

**Fig 1:**
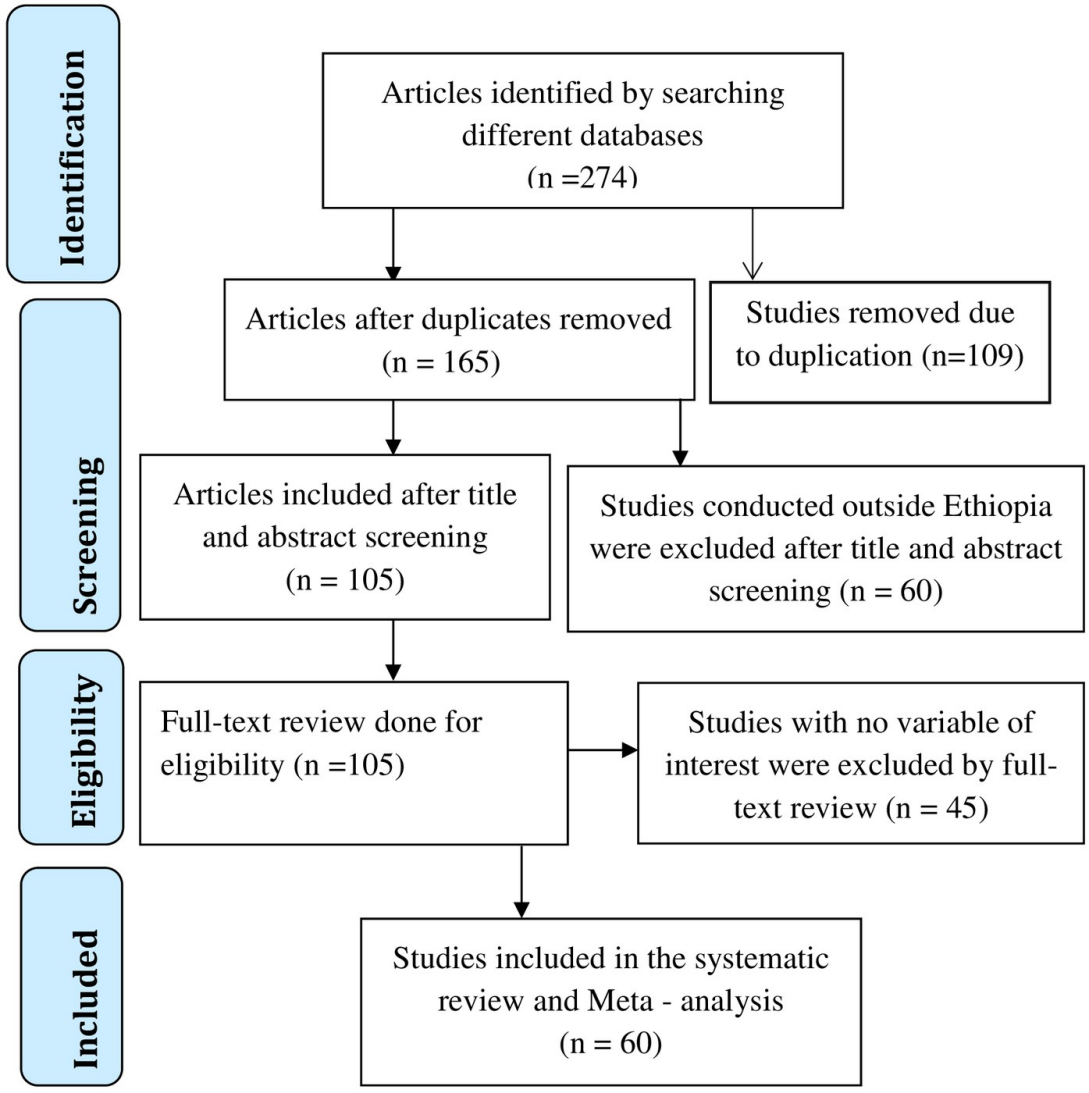
Flow chart on the article selection strategy for systematic review and meta-analysis of prevalence and predictors anemia among pregnant women in Ethiopia, 2021.

After search on the selected databases, 274 articles were found. One hundred and sixty-five articles were left after duplications had removed. These 165 articles were further reviewed by title and abstract, leading to the removal of 60 articles conducted outside Ethiopia. The 105 articles that were left had a full article review, at the end of which 45 articles were removed because they did not contain the variables of interest. Finally, 60 studies were included for the current systematic review and meta-analysis for prevalence and/or associated factors analysis as seen in the flow chart (Fig 1).

### Study characteristics

Among 60 included studies, 53 had cross-sectional designs, 5 were case-control studies, 2 were prospective cohort studies and one was a retrospective cohort study. The studies were done in different regions of Ethiopia; 6 in Tigray, 15 in Oromia, 14 in SNNPR, 16 in Amhara and 4 studies in the Addis Ababa city administration. There were; 2 studies in the Somalia region, one in Gambella, one study in Benshangul Gumuz and 1 was in Harari region. All the studies were published between 2010 and 2020, with sample sizes ranging from 150 [22] to 4600 participants [23]. Fifty four studies were considered in estimating the pooled prevalence of anemia with a total sample size of 28,542 (Table 1).

**Table 1:** Characteristics of studies included in the current systematic review and meta-analysis on prevalence and predictors of anemia among pregnant women in Ethiopia, 2021.

Author, Publication year	Study region	setting	Study design	Sample size	Prevalence of anemia
Addis, *et al*. (2014) [24]	Somalia, Gode	Community	Cross-sectional	581	56.8
Getachew, *et al*. (2012) [25]	SNNPR, Gilgel Gibe	Community	Cross-sectional	388	53.9
Mihiretie, *et al*. (2015) [22]	Oromia, Nekmite	Institution	Cross-sectional	150	50
Melku, M. and A. Agmas (2015) [26]	Amhara, Bahir Dar	Institution	Retrospective	1120	45.4
Kedir, *et al*. (2013) [27]	Harari, Haramaya	Community	Cross-sectional	1678	43.9
Gedefaw, *et al*. (2015) [28]	SNNPR,Woliata Sodo	Institution	Cross-sectional	363	39.9
Gari, *et al*. (2020) [29]	Oromia, Najo	Institution	Cross-sectional	384	37.8
Obse, *et al*. (2013) [30]	Oromia, Arsi	Institution	Cross-sectional	374	36.6
Beyene, T. (2018) [31]	Oromia, Arsi	Institution	Cross-sectional	374	36.6
Alemayehu, *et al*. (2016) [32]	Gambella, Pugnido	Institution	Cross-sectional	360	36.1
Gebre, *et al*. (2015) [33]	Tigray, North Western Zone	Institution	Cross-sectional	714	36.1
Geleta, W. and Z. Babure (2020) [34]	Oromia, Wollega	Community	Cross-section	625	35.5
Nega, *et al*. (2015) [35]	SNNPR, Arbaminch	Institution	Cross-sectional	341	34.6
Bekele, *et al*. (2016) [36]	SNNPR, Arbaminch	Institution	Cross-sectional	332	32.9
Helion, *et al*. (2020) [37]	Amhara, Gonder	Institution	Cross-sectional	713	32.4
Abay, *et al*. (2017) [38]	Beneshangul Gumuz, Asosa	Institution	Cross-sectional	761	31.8
Gebremariam, *et al*. (2020) [39]	SNNPR, Butajira	Institution	Cross-sectional	208	31.7
Gebremedhin, *et al*. (2014) [40]	SNNPR, Sidama	Community	Cross-sectional	700	31.6
Kenea, *et al*. (2018) [41]	Oromia, Ilu Abba Bora	Institution	Cross-sectional	416	31.5
Bolka, A. & S. Gebremedhin (2019) [42]	SNNPR, Sidama	Institution	Cross-sectional	352	31.5
Derso, *et al*. (2017) [43]	Amhara, Gonder	Institution	Cross-sectional	348	30.5
Ejeta, *et al*. (2014) [44]	Oromia, Nekemte	Institution	Cross-sectional	286	29
Argaw, *et al*. (2020) [45]	SNNPR, Dilla	Institution	Cross-sectional	373	28.7
Zerfu, *et al*. (2019) [46]	Oromia, Arsi	Institution	Cross-sectional	432	28.6
Mohammed, *et al*. (2018) [47]	Oromia, Adama	Institution	Cross-sectional	424	28.1
Kefiyalew, *et al*. (2014) [48]	Oromia, East Harerege	Institution	Cross-sectional	258	27.8
Getahun, *et al*. (2017) [49]	SNNPR, Butajira	Institution	Cross-sectional	217	27.6
Mulugeta, *et al*. (2020) [50]	SNNPR, Halaba Kulito	Institution	Cross-sectional	236	27.5
Belay, *et al*. (2018) [51]	Tigray, Mekele	Institution	Cohort	196	25.5
Asrie, F. (2017) [52]	Amhara	Institution	Cross-sectional	206	25.2
Delil, *et al*. (2018) [53]	SNNPR, Hossan	Institution	Cross-sectional	314	24.2
Zillmer, *et al*. (2017) [54]	Oromia	Community	Cohort	4600	24.09
Lebso, *et al*. (2017) [17]	SNNPR, Sidama	Community	Cross-sectional	507	23.2
Worku Takele, W. and A. Tariku (2018) [55]	Amhara, Gonder	Institution	Cross-sectional	362	22.2
Walelign, *et al*. (2018) [56]	Oromia, East Harerege	Institution	Cross-sectional	409	20.8
Umuro, *et al*. (2020) [57]	Addis Ababa, Tikur Ambessa Hospital	Institution	Cross-sectional	405	19.8
Abriha, *et al*. (2014) [16]	Tigiray, Mekele	Institution	Cross-sectional	619	19.7
Gudeta, *et al*. (2019) [58]	SNNPR, Benchimaji, Kefa, Shaka	Institution	Cross-sectional	1871	19
Samuel, *et al*. (2020) [59]	SNNPR, kambata Tabaro	Institution	Cross-sectional	423	18
Getaneh, *et al*. (2018) [60]	Amahara, Bahir Dar	Institution	Cross-sectional	480	18.3
Kejela, *et al*. (2020) [61]	Oromia, Wollega	Institution	Cross-sectional	286	17.8
Mengist, *et al*. (2017) [62]	Oromia, Wollega	Institution	Cross-sectional	372	17.5
Grum, *et al*. (2018) [63]	Tigray, Central zone	Institution	Cross-sectional	634	16.88
Melku, *et al*. (2014) [64]	Amhara, Gonder	Institution	Cross-sectional	302	16.6
Kebede, *et al*. (2018) [65]	Tigray, Shire	Institution	Cross-sectional	480	16.8
Ayano, *et al*. (2018) [66]	Oromia, Adama	Institution	Cross-sectional	329	14.9
Enawgaw, *et al*. (2019) [67]	Amhara, Gonder	Institution	Cross-sectional	217	12.9
Gebreweld, *et al*. (2018) [68]	Addis Ababa, St. paul’s hospital	Institution	Cross-sectional	284	11.62
Kumera, *et al*. (2018) [69]	Amhara, Debre Markos	Institution	Cross-sectional	234	11.5
Hailu, *et al*. (2019) [70]	Amhara, Gojam	Institution	Cross-sectional	743	10.6
Mekonnen, *et al*. (2018) [71]	Amhara, North Shoa	Institution	Cross-sectional	295	10
Berhe, *et al*. (2019) [72]	Tigray, Adigrat	Institution	Cross-sectional	304	7.9
Nasir, *et al*. (2020) [73]	Addis Ababa, Tikur Anbessa Hospital	Institution	Cross-sectional	250	4.8
Shitie, *et al*. (2018) [74]	Amhara, North Shoa	Institution	Cross-sectional	286	2.8
Feleke, B. E. and T. E. Feleke (2018) [75]	Amhara,Bahir Dar	Institution	Cross-sectional	550	
Tulu, *et al*. (2019) [14]	Oromia, Wollega	Institution	case-control	573	
Weldekidan, *et al*. (2018) [76]	SNNPR, Durame	Institution	case-control	333	
Osman, M. O. and T. Y. Nour (2020) [77]	Somalia, Jigjiga	Institution	case-control	228	
Tadesse, *et al*. (2017) [78]	Amhara, Eshete	Institution	case-control	448	
Mohammed, *et al*. (2019) [79]	Addis Ababa	Institution	Case-control	592	

### Prevalence of anemia among pregnant women

**Fig 2:**
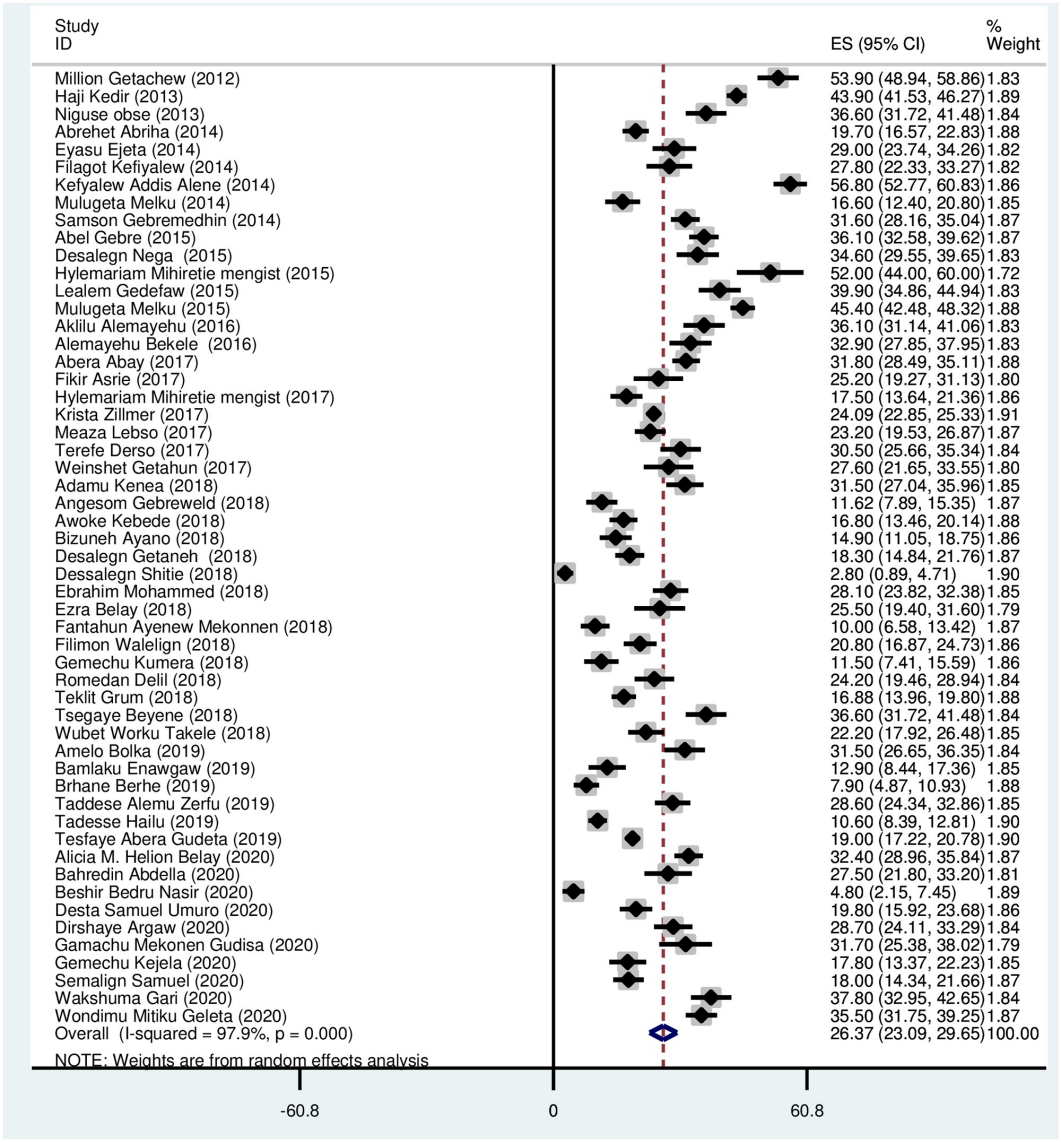
Forest plot on the prevalence of anemia among pregnant women in Ethiopia.

Prevalence of anemia among all the pregnant women pooled from fifty four studies was 26.37 (95% CI: 23.09, 29.65). Random effect analysis was done due to the presence of significant heterogeneity between studies (I^2^ = 97.9%, *p* < 0.001) as shown Fig 2. The lowest prevalence reported was 2.80 [74] and the highest prevalence was 56.80 [24] (Fig 2).

#### Subgroup analysis

Due to significant heterogeneity between studies, subgroup analysis has been done based on regions and study settings. This showed that the prevalence of anemia among pregnant women was 10.09% (95% CI: 25.67, 27.37) in Addis Ababa and 56.8% (52.77, 60.83) in the Somalia region. Sub-group analysis by the study settings showed higher pooled prevalence of 31.18, (95% CI: 30.27, 32.09) from community-based studies versus 20.37 (95% CI: 19.83, 20.92) in institution-based studies (Table 2).

**Table 2:** Subgroup analysis on the prevalence of anemia among pregnant women in Ethiopia.

Subgroups	No of study	I-V pooled ES			Heterogeneity	
Study setting		ES	CI 95%	% weight	I^2^	P-value
Institutional based study	46	20.37	19.83, 20.92	73.80	97.2	< 0.001
Community based study	8	31.18	30.27,32.09	26.20	98.6	< 0.001
**Regions**	**No of study**	**ES**	**CI 95%**	**% weight**	**I** ^**2**^	**P-value**
Oromia	14	26.52	25.67,27.37	29.93	93.6	< 0.001
Amhara	14	18.00	17.01,18.89	27.15	98.7	< 0.001
SNNPR	13	25.01	23.95,26.07	19.29	91.7	< 0.001
Tigray	6	18.94	17.56,29.32	11.43	96.7	< 0.001
Addis Ababa	3	10.09	8.21,11.98	6.11	95.0	< 0.001
Gambella	1	36.1	31.14,41.06	0.88	-	-
Somalia	1	56.8	52.77,60.83	1.34	-	-
Harari	1	43.9	41.52,46.27	3.86	-	-
Benshangul Gumuz	1	31.8	27.67 34.89	1.78	-	-

### Predictors of anemia

The systematic review and meta-analysis demonstrated that factors such as socio-demographic characteristics, reproductive history, dietary factors, chronic diseases and other medical conditions were associated with anemia among pregnant women in Ethiopia.

#### Socio-demographic factors

The association of socio-demographic factors such as residence, women’s education, marital status, and family size with maternal anemia was statistically significant. Studies showed that women resident in rural areas had a higher risk of being anemic than those from urban areas [41, 59, 70, 77, 80]. However, other studies included in systematic review showed there is no significant association between the residence and occurrence of anemia during pregnancy [14, 31, 34, 76] and other findings showed women from rural residents were less likely to develop anemia during pregnancy [25, 81]. Pooled risk ratio from 28 studies, that have reported this variable, showed women residing in rural place were more likely (RR = 1.56, 95% CI: 1.31, 1.86) to develop anemia than those living in urban areas. The random effect analysis was used due to significant heterogeneity (p < 0.001). The Begg’s and Egger’s tests for small-study effects showed there is no statistically significant publication bias (*p = 0*.*123*, *p = 0*.*284* respectively) as seen in Table 3.

**Table 3:** Association of socio-demographic factors with anemia among pregnant women in Ethiopia.

Factors	Number of studies	Pooled RR	95% CI	Heterogeneity		Publication bias	
				I^2^ (%)	p-value	Begg’s	Egger’s
Residence (rural)	28	1.56	1.31,1.86	88.8	< 0.001	0.123	0.284
Education (illiterate)	33	1.51	1.36,1.68	69.7	< 0.001	0.525	0.450
Occupation (housewife)	18	1.14	0.93,1.4	82.5	< 0.001	0.596	0.645
Family size(> = 5)	15	1.62	1.33,1.96	86.0	< 0.001	0.276	0.067
Marital status (married)	15	0.63	0.42,0.97	93.9	< 0.001	0.661	0.730

The pooled risk ratio from 33 studies, that reported educational level, showed the illiterate women were more likely (RR = 1.51, 95% CI: 1.36, 1.68) to be anemic than their counterparts. The heterogeneity test showed statistically significant heterogeneity between studies (I^2^ = 69.7, *p*< 0.001), and the Begg’s and Egger’s tests showed no significant publication bias (*p* = 0.525 and 0.450 respectively).

The pooled risk ratio on association of women’s occupation with anemia from 18 studies that reported the variable, it was found to be statistically insignificant. Women living with a family of 5 and more members were more likely (RR = 1.62, 95% CI: 1.33, 1.96) to be anemic than women living with lesser family members. Married women were 63% less likely to develop anemia than unmarried women (Table 3).

#### Obstetric factors

The meta-analysis showed that obstetric factors such as birth interval, gestational age, blood loss during pregnancy, antenatal clinic (ANC) follow up and history of excess menstrual bleeding were significantly associated with anemia during pregnancy(*p* < 0.001).

Birth interval less than 24 months had a higher risk for anemia on pregnancy than an interval greater than 24 months [14, 31, 34, 82]. In contrast to this, other studies reported birth interval had no statistically significant association with maternal anemia [62, 66, 74, 81, 83]. However, pooled risk ratio showed women who got pregnant within 24 months birth interval were more likely (RR = 1.55(95% CI; 1.31,1.83)) to be anemic than their counterparts. Due to statistically significant heterogeneity between studies, random effect analysis was used (Table 4).

**Table 4:** Association of obstetric history and anemia among pregnant women in Ethiopia.

Factors	Number of study	Pooled RR	95% CI	Heterogeneity		Publication Bias	
				I^2^ (%)	p-value	Begg’s	Egger’s
Gravidity(multi-gravidity)	26	1.10	0.97,1.24	79.5	< 0.001	0.630	0.774
Birth interval(< 24 month)	26	1.55	1.31,1.83	76.0	< 0.001	0.643	0.404
Gestational age(3^rd^ trimester)	34	1.19	1.01,1.32	80.4	< 0.001	0.343	0.001
Blood loss in current pregnancy	5	2.21	1.79,2.72	20.7	0.283	0.462	0.326
ANC follow up (no)	10	1.36	1.04,1.80	92.7	< 0.001	0.095	0.755
Excess menstrual bleeding	8	1.86	1.35,2.57	89.3	< 0.001	0.048	0.054

The pooled risk ratio from 34 studies showed women in the third trimester had a higher risk of anemia (RR = 1.19, (95% CI; 1.01, 1.32)) than those women in the first and second trimester. Women with no history of ANC visit were more likely (RR = 1.36, (95% CI; 1.04, 1.80)) to be anemic than those at ANC follow up. Women with a history of blood loss during pregnancy were nearly two times more at a risk of being anemic than those with no history of blood loss. History of excess menstrual bleeding is also positively associated with anemia during pregnancy. Random effect analysis was used due to significant heterogeneity between studies, and Begg’s and Egger’s analysis showed there was no statistically significant publication bias as shown in Table 4.

#### Dietary factors

**Fig 3:**
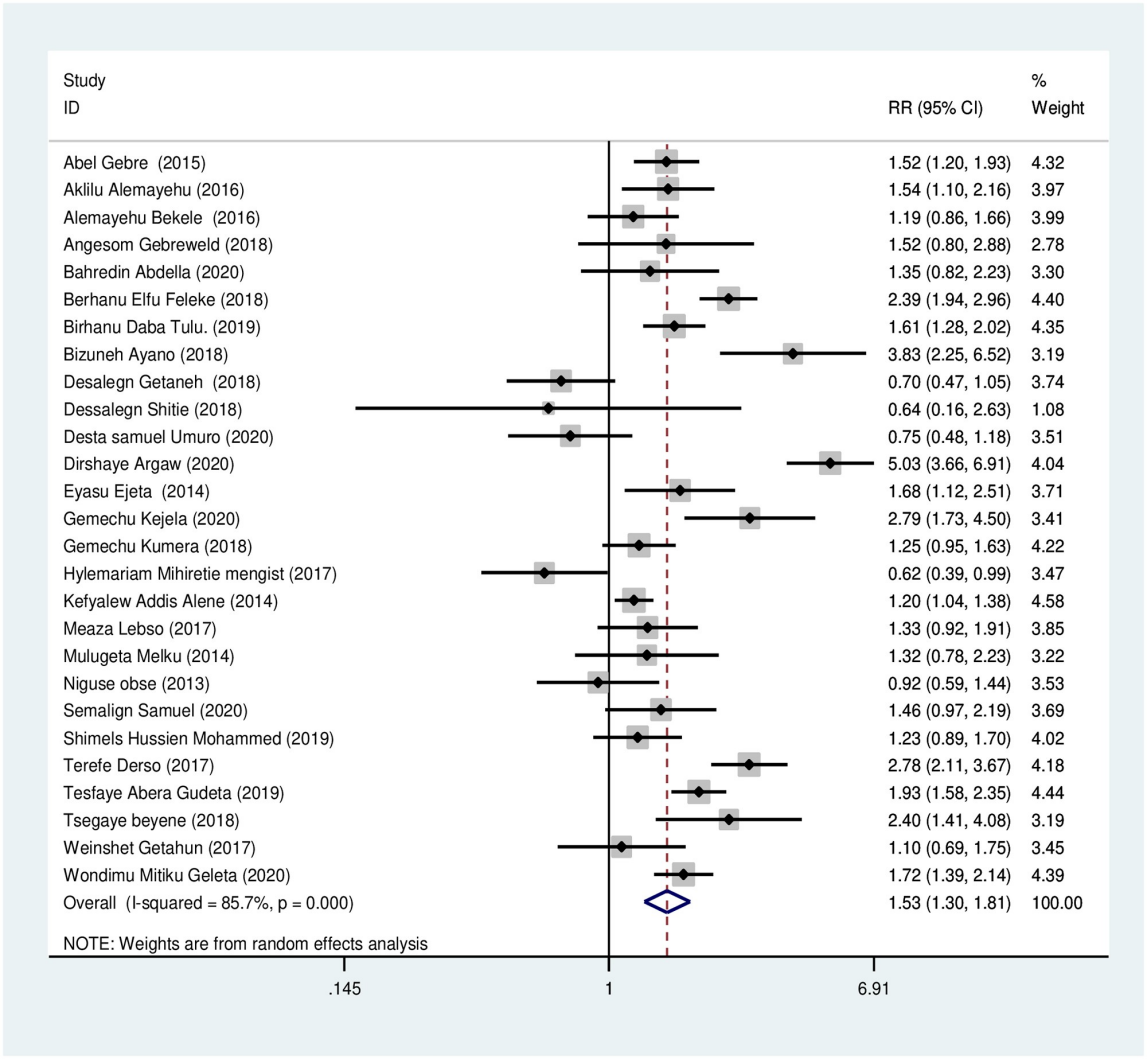
Association of iron-folic acid supplementation with anemia during pregnancy.

*Iron and folic acid supplementation*. Women who did not take iron-folic acid during pregnancy were 1.53 times more likely to be anemic during pregnancy than those who took iron-folic acid regularly, RR = 1.53 (95% CI, 1.30, 1.81). Due to the presence of significant heterogeneity (I^2^ = 85.7%, P< 0.001), random effect analysis has been used. Begg’s and Egger’s tests showed no statistically significant evidence for publication bias (*p* = 1.00, *p* = 0.863 respectively) (Fig 3).

**Fig 4:**
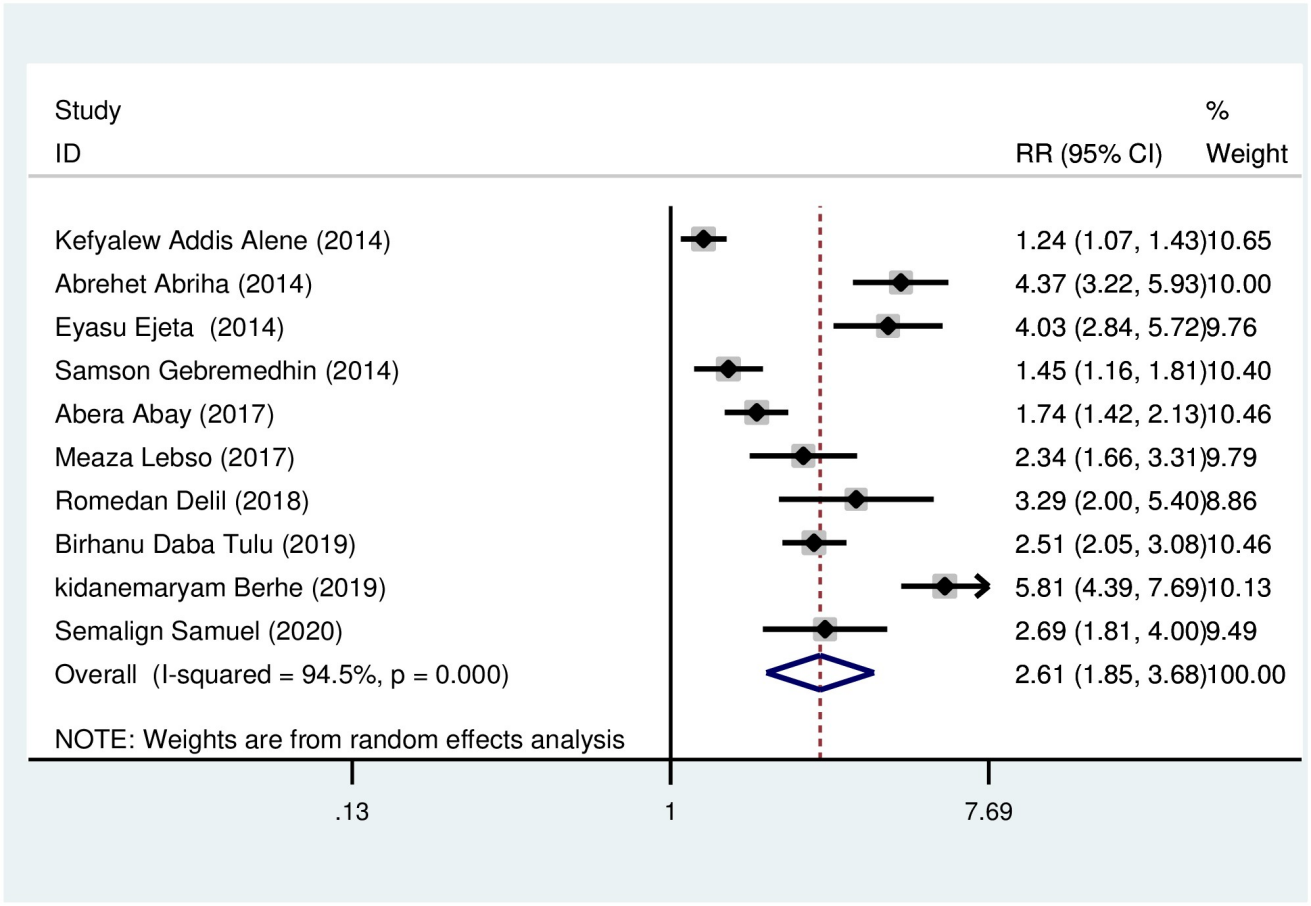
Association of low dietary diversity with anemia during pregnancy.

*Low dietary diversity*. Women with low dietary diversity were more likely (RR = 2.61, 95% CI: 1.97, 3.48) to develop anemia during pregnancy in comparison to those women taking medium and high dietary diversity. The heterogeneity test showed statistically significant heterogeneity (I^2^ = 94.5, *p* < 0.001) (Fig 4). In testing for publication bias, the Begg’s and Egger’s tests showed no statistically significant evidence of small study effect, *p* = 0.107 and 0.060 respectively.

**Fig 5:**
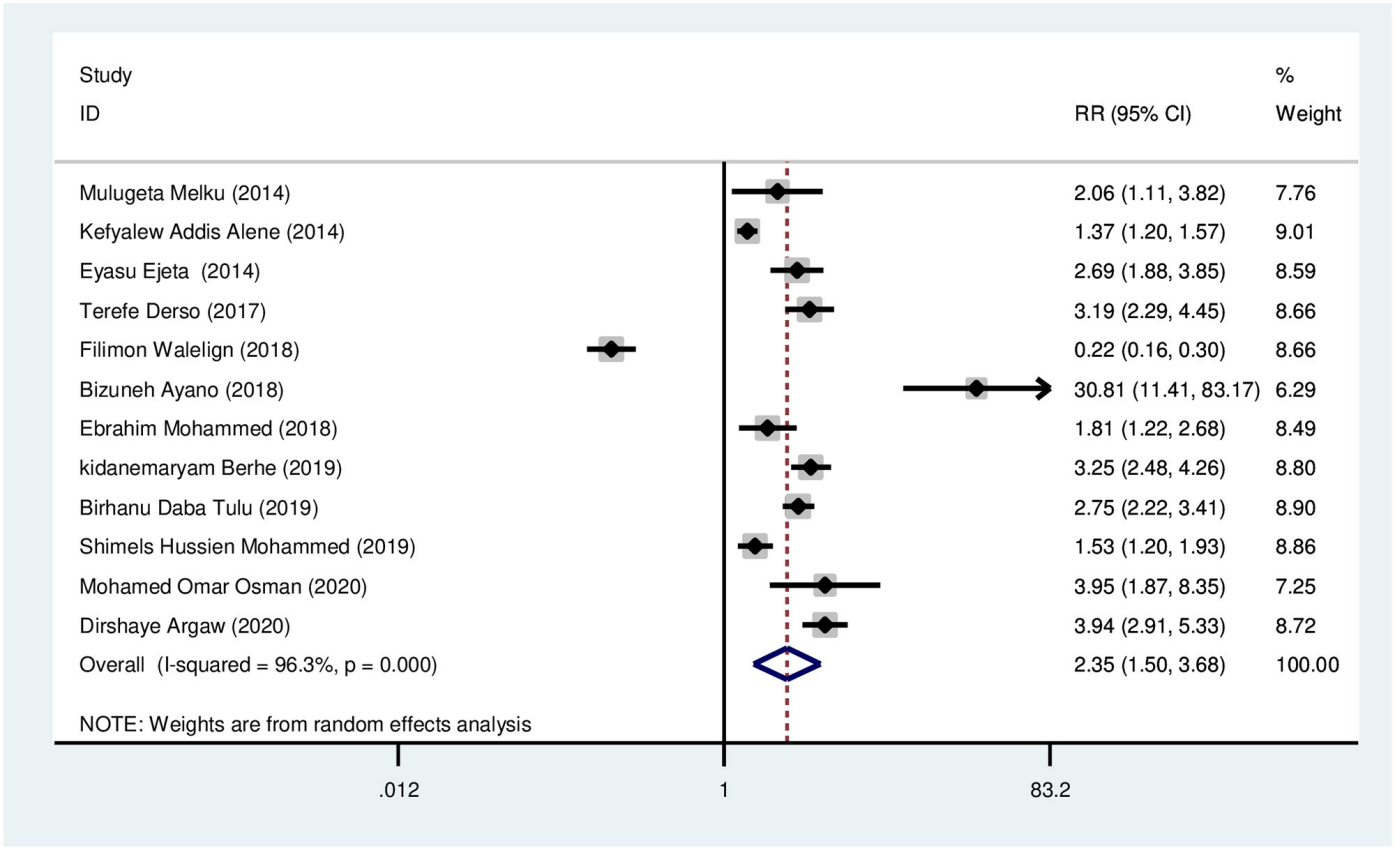
Association of mid-upper arm circumference with anemia during pregnancy.

*Mid upper arm circumference (MUAC)*. Meta-analysis showed women with low mid-upper arm circumference (MUAC < 23) were 2.35 times more likely to develop anemia during pregnancy in comparison to their counterparts. There was significant heterogeneity between the studies (I^2^ = 96.3%, *p* < 0.001) as seen in Fig 5. Begg’s and Egger’s tests for publication bias showed there was no statistically significant evidence of small study effect, *p* = 0.631 and 0.677 respectively.

#### Medical conditions

Medical conditions such as malaria infection, intestinal parasites, and HIV infection were statistically significantly associated with anemia during pregnancy. It has been shown that there was statistically significant heterogeneity between the studies, and Begg’s and Egger’s test showed statistically insignificant evidence for publication bias Table 5.

**Table 5:** Association of the different medical condition with anemia during pregnancy.

Factors	Number of studies	Pooled RR	95% CI	Heterogeneity		Publication Bias(*p*)	
				I^2^ (%)	p-value	Begg’s	Egger’s
HIV infection (+ve)	11	2.43	1.94, 3.05	74.2	< 0.001	0.592	0.916
Other chronic disease	5	1.54	0.93, 2.53	85.1	< 0.001	0.806	0.155
Malaria	16	1.96	1.55, 2.48	82.3	< 0.001	0.192	0.102
Intestinal parasites	23	2.18	1.66, 2.87	93.3	< 0.001	0.635	0.549

## Discussion

The systematic review and meta-analysis estimated pooled prevalence of anemia among pregnant women in Ethiopia to be 26.37 (95% CI: 23.09, 29.65). It also revealed factors associated with anemia among pregnant women. This will help policymakers and program planners to take appropriate action to solve the problem among this vulnerable group.

According to the WHO, anemia is a health condition of moderate public health significance [2]. Prenatal anemia continues to be a problem among pregnant women adding to maternal morbidities such as gestational hypertension, pre-eclampsia, and obstructed labor [11, 12]. Unless attention is given, it will pose a threat to the success of reducing adverse maternal and fetal outcomes.

The current report on the prevalence of anemia is nearly comparable with a similar study done in Uganda (prevalence of25.8%) [84]. On the other hand the prevalence of anemia in this study is lower than studies conducted using nationwide data from Ethiopia (prevalence of 41%); 2016 EDHS [7] and meta-analysis conducted in 2017 (prevalence of31.66%) [15]. The above difference from national database may be due to inclusion of more institutional based studies at which prevalence of anemia is lower than in community setting. It could also be due to incorporation of low prevalence reports from most recent studies. The result of current study is also lower than the report of studies from other countries; 40.8% in Ghana, 53% in Sudan, and 54.5% in Nigeria [85–87]. This may be attributed to a difference in participant’s socio-demographic background, maternal health services, and factors that determine the nutrition and wellbeing of women.

Sub-group analysis of this study showed pooled prevalence from eight community-based studies was 31.18 (30.27, 32.09). This was much higher than pooled prevalence from 46 institutional-based studies; 20.37 (19.83, 20.92). This difference may be due to the participants of institutional-based studies were ANC followers that are exposed to health education and receive iron-folic acid supplementation in addition to other measures that promote their health, unlike participants of community-based studies in which some did not attend ANC. This finding in line with another study that revealed a high prevalence of anemia in community-based studies compared to institution-based studies [15]. The pooled prevalence of anemia from institutional-based studies (20.37%) is much lower than the pooled prevalence of anemia from institutional-based studies in Sudan (53.0%) [87]. Subgroup analysis based on the region also showed the lowest prevalence of anemia in Addis Ababa (10.09%) and a high prevalence of anemia in the Somalia region (56.8%). This difference in prevalence may be attributed to the difference in socio-demographic status, iron folate intake, and availability of health services [15].

The current study revealed socio-demographic factors were significantly associated with anemia during pregnancy. The meta-analysis showed women from rural areas and those who are illiterate were more likely to be anemic than their counterparts. This could be due to differences in socioeconomic status, weak access to health services in rural areas, and inadequate access to a variety of food items in rural communities. Educated women have more awareness on role of iron supplementation, seek out health services and include food choices that favor decreased risk of anemia. This has also been seen in studies conducted and Pakistan were women’s education level and region of residence was significantly associated with anemia during pregnancy [88, 89].

Family size was found to be significantly associated with anemia, with> = 5 family members leading to a 1.62 times increased risk of anemia in women from these households. This could be due to larger family sizes resulting in household food insecurity and decreased intake of nutrients that could have prevented anemia. Another study had also reported larger family size was associated with anemia during pregnancy [89].

The analysis showed married women were 63% less likely to develop anemia than unmarried women. This may be due to spousal support during pregnancy including economic support, ensuring ANC attendance and the wife’s adherence to the instruction of health professionals, thereby ensuring the overall well-being of pregnant women and reduce the risk of anemia. This is an observation that has also been made by others [90].

The study showed women with a history of birth interval less than 24 months and at their third trimester were more likely anemic than their counterparts. Association of a short birth interval with maternal anemia may be due to lack of enough time to restore micronutrient storage. This report in line with another study conducted in Bangladesh [91]. Women in the third trimester were also more likely to be anemic. This may be due to increased demand for iron as pregnancy advances coupled with the exhaustion of iron stores in most women. A study in northern Ghana also showed that pregnant women in the third trimester were about four times more likely to be anemic compared to those in the first and second trimester [92].

Women with a history of ANC follow-up were less likely to be anemic in comparison with women with no ANC visit at all. This may be due to health education provided at ANC visits [53], and access to health care services like iron-folic acid supplementation during such visits. The analysis showed women not taking iron-folic acid supplementation were 1.53 times more likely anemic than their counterparts. This is in line with a study done in northern India [93]. On the other hand adherence to iron-folic acid supplementation depends on ANC uptake which in turn is directly associated with anemia.

The nutritional status of women was statistically significantly associated with maternal anemia. Women with a MUAC less than 23 cm, which indicates overall poor nutritional status, were 2.35 more likely to be anemic than their counterparts, which is in agreement with previous report from other places [94, 95].

The meta-analysis showed low maternal dietary diversity was statistically positively associated with maternal anemia. Women who consumed a low diversified diet were 2.61 times more likely to be anemic than women who ate at least a medium diversified diet, in line with findings from other places such as Ghana [96, 97]. Pregnancy is a period in a woman’s life in which there is a high demand for nutrients. Hence, as the diversity of diet consumed decreases, the number, and quality of micronutrient adequacy decreases which may result in nutritional anemia due to the unmet need for iron and other micronutrients. It is noteworthy that in contrast to this, other studies from the rural areas of Ghana and Pakistan showed there was no association between dietary diversity and maternal anemia [98, 99].

The relationship between dietary diversity and maternal anemia is yet to be fully defined, particularly in places where many factors other than dietary diversity contribute to the risk of anemia [98]. However, dietary diversity is found to be a key indicator for utilization and quality of diet at the individual level and a proxy measure for adequacy of nutrient intake during pregnancy [100]. A pregnant woman’s diet that lacks diversity is most likely to be deficient in important nutrients such as iron, folate, and vitamin B12 which may lead to anemia [101]. A study from Kenya showed dietary diversity had a significant positive linear relationship with maternal anemia [102].

Women with a history of malaria infection during pregnancy were nearly two times more likely to be anemic in comparison with women with no history of malaria infection. This agrees with finding from Ghana, Kenya, Tanzania, and Sudan [87, 103, 104]. Malaria parasitemia is known to lyse erythrocyte, leading to anemia. Intestinal parasitic infection during pregnancy is also associated with maternal anemia in agreement with previous studies done in Kenya and Nigeria [105, 106].

The meta-analysis showed HIV infection was significantly associated with anemia during pregnancy. The HIV-positive women had more than two times higher risk of having anemia than their HIV- negative counterparts, a finding that had also been previously reported from South- eastern Nigeria [107]. The association between HIV infection and anemia can be explained in multiple ways. HIV infection may cause anemia, probably as a consequence of HIV infection of stromal cells and bone marrow suppression by antiretroviral therapy that results in decreased red blood cell production [108].

The primary studies included in this meta-analysis were searched intensively and critical appraisal for each study was done by using the Joanna Briggs Institute Meta-Analysis of Statistics Assessment and Review Instrument (JBI-MAStARI). The PRISMA guideline was strictly followed during the review processes. Almost all the variables included in the primary studies were addressed for analysis. However, the study has some limitations. Firstly, due to cross-sectional nature of most studies causal relationships and confounding factors cannot be controlled. Most of the studies were from three regions; Amhara, SNNPR, and Oromia with only a few studies from other regions. For example only one study from the Somalia region, Gode town with the highest prevalence (56.8%) that may not be representative of the whole region. Some studies did not consider the altitude of residents in measuring hemoglobin level.

## Conclusion

The meta-analysis on the prevalence of anemia showed almost one in four pregnant women in Ethiopia had anemia. Socio-demographic factors such as place of residence, women’s education, family size, and marital status had statistically significant association with maternal anemia. Anemia during pregnancy also had a significant association with reproductive and obstetric history like birth interval, gestational age, ANC follow-up, and blood loss during pregnancy. Factors related to nutritional status such as MUAC, iron folate supplementation, and maternal dietary diversity were found to be significantly associated with anemia. Comorbidities like malaria infection, intestinal parasites, and HIV infection are also associated with maternal anemia.

The responsible bodies; governmental and non-governmental organizations, should give due focus to women with high risk. Health professionals at facilities and health extension workers at the community level should work on long-acting family planning to increase birth spacing and they should mobilize women for proper utilization of ANC service. Health education regarding iron foliate intake and necessary support to improve dietary diversity should be given to pregnant women. Government should give attention to pregnant women on the prevention of malaria by providing insecticide treated net (ITN), and early diagnosis, and appropriate treatment. WHO guidelines for deworming for pregnant women should be ensured. Ultimately, further longitudinal studies set up to include subjects across the different variables that contribute to maternal anemia are needed.

## Supporting information

S1 File Declarations. (DOCX)

S2 File PROSPERO published protocol. (PDF)

S3 File Publication bias or risk assessment. (DOCX)

S1 Checklist PRISMA 2009 checklist. (DOC)
