# Supplementary material for: Prevalence and predictors of anemia among pregnant women in Ethiopia: Systematic review and meta-analysis
Source: PLoS One. 2022 Jul 27;17(7):e0267005. doi: 10.1371/journal.pone.0267005 (PMC9328503; doi:10.1371/journal.pone.0267005)
Supplement: S1 File — (DOCX) [file pone.0267005.s001.docx]

**Declarations**

**Authors' Contributions**

All authors made a significant contribution to the work reported, whether that is in the conception, study design, execution, acquisition of data, analysis and interpretation, or in all these areas; took part in drafting, revising or critically reviewing the article; gave final approval of the version to be published; have agreed on the journal to which the article has been submitted; and agree to be accountable for all aspects of the work.

**Competing Interests**

The authors have declared that they have no competing interests exist

**Financial Disclosure**

This study was supported by the Pan African University (PAU), a continental initiative of the African Union Commission (AU), Addis Ababa, Ethiopia, as part of the Ph.D. program. Teshome received funding from PAU. The University had no role in the study design, data collection and analysis, decision to publish, or preparation of the manuscript
