# Supplementary material for: Prevalence and predictors of anemia among pregnant women in Ethiopia: Systematic review and meta-analysis
Source: PLoS One. 2022 Jul 27;17(7):e0267005. doi: 10.1371/journal.pone.0267005 (PMC9328503; doi:10.1371/journal.pone.0267005)
Supplement: S2 File — (PDF) [file pone.0267005.s002.pdf]

Systematic review

This record cannot be edited because it is being assessed by the editorial team

1. * Review title.

Give the title of the review in English

Prevalence and predictors of anemia among pregnant women in Ethiopia: systematic review and meta-analysis

2. Original language title.

For reviews in languages other than English, give the title in the original language. This will be displayed with the English language title.

3. * Anticipated or actual start date.

Give the date the systematic review started or is expected to start.

24/09/2020

4. * Anticipated completion date.

Give the date by which the review is expected to be completed.

24/11/2020

5. * Stage of review at time of this submission.

Tick the boxes to show which review tasks have been started and which have been completed.
Update this field each time any amendments are made to a published record.

Reviews that have started data extraction (at the time of initial submission) are not eligible for inclusion in PROSPERO.
If there is later evidence that incorrect status and/or completion date has been supplied, the published PROSPERO record will be marked as retracted.

This field uses answers to initial screening questions. It cannot be edited until after registration.

The review has not yet started: No

Review stage	Started	Completed
Preliminary searches	Yes	No
Piloting of the study selection process	Yes	No
Formal screening of search results against eligibility criteria	Yes	No
Data extraction	No	No
Risk of bias (quality) assessment	No	No
Data analysis	No	No

Provide any other relevant information about the stage of the review here.

6. * Named contact.

The named contact is the guarantor for the accuracy of the information in the register record. This may be any member of the review team.

Teshome Gensa Geta

Email salutation (e.g. "Dr Smith" or "Joanne") for correspondence:
Mr Geta

7. * Named contact email.

Give the electronic email address of the named contact.

teshgen2006@gmail.com

8. Named contact address

PLEASE NOTE this information will be published in the PROSPERO record so please do not enter private information, i.e. personal home address

Give the full institutional/organisational postal address for the named contact.

P.O.BOX: 07

9. Named contact phone number.

Give the telephone number for the named contact, including international dialling code.

+251- 910686501
+234-7067646813

10. * Organisational affiliation of the review.

Full title of the organisational affiliations for this review and website address if available. This field may be completed as 'None' if the review is not affiliated to any organisation.

Pan Africa University Life and Earth Science, Department of Obstetrics and Gynaecology, College of Medicine, University of Ibadan, Ibadan, Nigeria

College of Health Science and Medicine, Wolkite University, Wolkite, Ethiopia

Organisation web address:

11. * Review team members and their organisational affiliations.

Give the personal details and the organisational affiliations of each member of the review team. Affiliation refers to groups or organisations to which review team members belong.
NOTE: email and country now MUST be entered for each person, unless you are amending a published record.

Mr Teshome Gensa Geta. 1. Pan African University Life and Earth Science Institute, University of Ibadan, Ibadan, Nigeria. 2. College of Health Science and Medicine, Wolkite University, Wolkite, Ethiopia

Professor Akinyinka OMIGBODUN. Pan African University Life and Earth Science Institute, Department of Obstetrics and Gynecology, College of Medicine, University of Ibadan, Ibadan, Nigeria

Dr Samson GEBREMEDHIN. School of Public Health, Addis Ababa University, Addis Ababa, Ethiopia

12. * Funding sources/sponsors.

Details of the individuals, organizations, groups, companies or other legal entities who have funded or sponsored the review.

no fund for the review

Grant number(s)

State the funder, grant or award number and the date of award

13. * Conflicts of interest.

List actual or perceived conflicts of interest (financial or academic).

None

14. Collaborators.

Give the name and affiliation of any individuals or organisations who are working on the review but who are not listed as review team members. **NOTE: email and country must be completed for each person, unless you are amending a published record.**

15. * Review question.

State the review question(s) clearly and precisely. It may be appropriate to break very broad questions down into a series of related more specific questions. Questions may be framed or refined using PI(E)COS or similar where relevant.

what is the prevalence of anemia among pregnant women in Ethiopia
what are the predictors of anemia among pregnant women in Ethiopia

16. * Searches.

State the sources that will be searched (e.g. Medline). Give the search dates, and any restrictions (e.g. language or publication date). Do NOT enter the full search strategy (it may be provided as a link or attachment below.)

The articles for this systematic review and meta-analysis will be searched by electronic databases such as PubMed/MEDLINE, google scholars, HINARI, ScienceDirect, AJOL, and EMBASE. Further tracing of studies will be done by direct contact with the corresponding author of the existing article through email.

The original research articles that reported the prevalence and predictors of anemia among pregnant women in different regions of Ethiopia and published until October 30, 2020, will be included. All accessible full-text articles written in the English language will be eligible with no restriction to study design.

17. URL to search strategy.

Upload a file with your search strategy, or an example of a search strategy for a specific database, (including the keywords) in pdf or word format. In doing so you are consenting to the file being made publicly accessible.

Or provide a URL or link to the strategy. Do NOT provide links to your search **results**.

https://www.crd.york.ac.uk/PROSPEROFILES/211054_STRATEGY_20200925.pdf

Do not make this file publicly available until the review is complete

18. * Condition or domain being studied.

Give a short description of the disease, condition or healthcare domain being studied in your systematic review.

Anemia is a condition of a reduced number of circulating RBCs or insufficiency of the oxygen-carrying capacity of RBC to meet physiologic needs. Hemoglobin concentration is the most common hematological assessment method used to define anemia. World health organization (WHO) defined anemia for pregnant women as a hemoglobin concentration less than 110gm/l at sea level.

The prevalence of anemia among pregnant women in low and middle-income countries is 38.9% to 48.7%. The Ethiopian Demographic Health Survey 2016 (EDHS) showed the prevalence of anemia among pregnant women was 41%. Anemia during pregnancy is an important predictor of poor pregnancy outcomes such as low birth weight (LBW), prematurity, and stillbirth. It is also associated with maternal morbidities such as abortions, antepartum hemorrhage, postpartum hemorrhage, preeclampsia, and prolonged labor.

Multiple factors contribute to the occurrence of anemia during pregnancy in addition to physiologic changes. Dietary patterns such as meal frequency and low dietary diversity score and nutritional status of women during pregnancy may be an important predictor of anemia. Other factors associated with anemia are low socioeconomic status, malaria, helminthic infection, small birth interval and late initiation of ANC follow up. All the factors work in the interaction with one another.

19. * Participants/population.

Specify the participants or populations being studied in the review. The preferred format includes details of both inclusion and exclusion criteria.

Inclusion criteria

All pregnant women included in the original studies irrespective of gestational age will be the population of the current review.

Exclusion criteria

Women whose information collected after delivery will be excluded.

20. * Intervention(s), exposure(s).

Give full and clear descriptions or definitions of the interventions or the exposures to be reviewed. The preferred format includes details of both inclusion and exclusion criteria.

Exposures(predictor variables)

□ Sociodemographic histories such as advanced age, rural residence, no formal education, unemployment, and household size

□ Obstetric and medical history such as low birth interval, being the third trimester, multigravida, no ANC uptake, malaria infection, helminthic infection, and HIV infection

□ Nutrition status of women i.e MUAC below standard, not taking iron foliate, less frequency of meal per day than usual, and low dietary diversity.

Other exposure variables will be included as they arise during the review, with justifications for their inclusion

21. * Comparator(s)/control.

Where relevant, give details of the alternatives against which the intervention/exposure will be compared (e.g. another intervention or a non-exposed control group). The preferred format includes details of both inclusion and exclusion criteria.

Comparative/ control groups for the current review will be women with better socioeconomic status i.e urban residence, having a formal education, getting employed than their counterparts. Women with the gestational age of first and second trimester, primigravida, women taking ANC service, women with no malaria, helminthic, and HIV infections will be also the control group. Moreover, pregnant women with normal MUAC measurement, took iron folate supplementation, women taking a frequent meal, and having high dietary diversity is considered in this group.

22. * Types of study to be included.

Give details of the study designs (e.g. RCT) that are eligible for inclusion in the review. The preferred format includes both inclusion and exclusion criteria. If there are no restrictions on the types of study, this should be stated.

no restriction to study design

23. Context.

Give summary details of the setting or other relevant characteristics, which help define the inclusion or exclusion criteria.

The setting of the included primary study can be either community based or health facilities

24. * Main outcome(s).

Give the pre-specified main (most important) outcomes of the review, including details of how the outcome is defined and measured and when these measurement are made, if these are part of the review inclusion criteria.

Prevalence of anemia among pregnant women
- anemia is defined as a hemoglobin concentration less than 110g/l at sea level.

predictors of anemia
- predictors considered for the current review will be socio-demographic factors, obstetric and medical factors, nutritional status, and dietary pattern.

* Measures of effect

To show the association between predictor variables and outcome variables i.e anemia, odds ratio (OR) with a 95% confidence interval will be used.

25. * Additional outcome(s).

List the pre-specified additional outcomes of the review, with a similar level of detail to that required for main outcomes. Where there are no additional outcomes please state ‘None’ or ‘Not applicable’ as appropriate to the review

none

* Measures of effect

none

26. * Data extraction (selection and coding).

Describe how studies will be selected for inclusion. State what data will be extracted or obtained. State how this will be done and recorded.

After fully addressing all the available articles, the duplicate will be removed using endnote. Then the title and abstract of all the studies will be reviewed. The full-text review will be done for relevant articles. Finally, articles with variables of interest will be included in the review.

The two authors will independently extract all necessary data by using Microsoft excel data extraction format. The data extraction format includes the author's name, year of publication, study design, sample size, and prevalence of anemia. The format will also include the prevalence of anemia among women with and without exposure. After completing data extraction, it will be checked for consistency and completeness. Then, the extracted data will be checked again by the three authors, and disagreement will be solved by tracing back to the original articles. Finally, incomplete data will be traced by direct contact with the principal author using an email address.

27. * Risk of bias (quality) assessment.

State which characteristics of the studies will be assessed and/or any formal risk of bias/quality assessment tools that will be used.

Quality of all the study will be assessed by critical appraisal using the Joanna Briggs Institute Meta-Analysis of Statistics Assessment and Review Instrument (JBI-MAStARI). This critical appraisal tool addresses the methodology of the study. It includes sample representativeness, sample adequacy, appropriate recruitment of participants, sufficient description of participants, use of standard measurement and its reliability, appropriate statistical analysis done with sufficient coverage of identified sample size, appropriate statistical analysis used, and subgroup or confounding variables identified. All the components of quality assessment will be discussed among the review team. The quality assessment of all articles will be done by two investigators independently. Any disagreement between them will be resolved by the negotiation of the third member of the review team. Studies with a score greater than or equal to 6 out of 10 will be considered as high quality and those studies with less than 6 out of 10 considered as low quality. Those studies with high quality will be included in the meta-analysis.

28. * Strategy for data synthesis.

Describe the methods you plan to use to synthesise data. This **must not be generic text** but should be **specific to your review** and describe how the proposed approach will be applied to your data.
If meta-analysis is planned, describe the models to be used, methods to explore statistical heterogeneity, and software package to be used.

Extracted data from Microsoft will be transported to STATA software version 14 for analysis. The general character of original articles such as the first author name, year of publication, study area, study setting, study design, and sample size will be presented using a table. The prevalence and its standard error of each original article will be considered to calculate the pooled prevalence of anemia. The pooled prevalence will be presented using a forest plot. The heterogeneity among the prevalence of anemia in the studies will be tested by the I^2 statistical test and a p-value less than 0.05 will be used to declare it. If there is

significant heterogeneity between the studies, a random effect meta-analysis model will be used. Moreover, a meta-regression model will be used to detect possible sources of heterogeneity. The potential publication bias will be assessed by using Egger's correlation test at a 5% significance level.

29. * Analysis of subgroups or subsets.

State any planned investigation of 'subgroups'. Be clear and specific about which type of study or participant will be included in each group or covariate investigated. State the planned analytic approach.

To reduce the random variation between point estimates of the primary study, subgroup analysis will be done based on different regions of the country.

30. * Type and method of review.

Select the type of review, review method and health area from the lists below.

Type of review

Cost effectiveness	No
Diagnostic	No
Epidemiologic	Yes
Individual patient data (IPD) meta-analysis	No
Intervention	No
Meta-analysis	Yes
Methodology	No
Narrative synthesis	No
Network meta-analysis	No
Pre-clinical	No
Prevention	No
Prognostic	No
Prospective meta-analysis (PMA)	No
Review of reviews	No
Service delivery	No
Synthesis of qualitative studies	No
Systematic review	Yes
Other	No

Health area of the review

Alcohol/substance misuse/abuse	No

Blood and immune system	No
Cancer	No
Cardiovascular	No
Care of the elderly	No
Child health	No
Complementary therapies	No
COVID-19	No
Crime and justice	No
Dental	No
Digestive system	No
Ear, nose and throat	No
Education	No
Endocrine and metabolic disorders	No
Eye disorders	No
General interest	No
Genetics	No
Health inequalities/health equity	No
Infections and infestations	No
International development	No
Mental health and behavioural conditions	No
Musculoskeletal	No
Neurological	No
Nursing	No
Obstetrics and gynaecology	No
Oral health	No
Palliative care	No
Perioperative care	No

Physiotherapy	No
Pregnancy and childbirth	Yes
Public health (including social determinants of health)	Yes
Rehabilitation	No
Respiratory disorders	No
Service delivery	No
Skin disorders	No
Social care	No
Surgery	No
Tropical Medicine	No
Urological	No
Wounds, injuries and accidents	No
Violence and abuse	No

31. Language.

Select each language individually to add it to the list below, use the bin icon to remove any added in error.

English

There is an English language summary.

32. * Country.

Select the country in which the review is being carried out. For multi-national collaborations select all the countries involved.

Ethiopia

33. Other registration details.

Name any other organisation where the systematic review title or protocol is registered (e.g. Campbell, or The Joanna Briggs Institute) together with any unique identification number assigned by them.
If extracted data will be stored and made available through a repository such as the Systematic Review Data Repository (SRDR), details and a link should be included here. If none, leave blank.

34. Reference and/or URL for published protocol.

If the protocol for this review is published provide details (authors, title and journal details, preferably in Vancouver format)

No I do not make this file publicly available until the review is complete

35. Dissemination plans.

Do you intend to publish the review on completion?

Yes

36. Keywords.

Give words or phrases that best describe the review. Separate keywords with a semicolon or new line. Keywords help PROSPERO users find your review (keywords do not appear in the public record but are included in searches). Be as specific and precise as possible. Avoid acronyms and abbreviations unless these are in wide use.

Prevalence, Predictors, Pregnant Women, Anemia, Ethiopia

37. Details of any existing review of the same topic by the same authors.

If you are registering an update of an existing review give details of the earlier versions and include a full bibliographic reference, if available.

38. * Current review status.

Update review status when the review is completed and when it is published.
New registrations must be ongoing.

Review_Ongoing

39. Any additional information.

Provide any other information relevant to the registration of this review.

40. Details of final report/publication(s) or preprints if available.

Leave empty until publication details are available OR you have a link to a preprint.
List authors, title and journal details preferably in Vancouver format.
