# Supplementary material for: Prevalence and predictors of anemia among pregnant women in Ethiopia: Systematic review and meta-analysis
Source: PLoS One. 2022 Jul 27;17(7):e0267005. doi: 10.1371/journal.pone.0267005 (PMC9328503; doi:10.1371/journal.pone.0267005)
Supplement: S3 File — (DOCX) [file pone.0267005.s003.docx]

**Publication bias**

Publication bias in this research was assessed using funnel plot, Egger’s and Begg’s test. The overall publication bias was done among fifty four studies; articles from which proportion of anaemia determined and also the bias among articles included for each predictor variable was assessed. The assessment on overall publication bias done from proportion showed presence of significant bias among 54 studies but there is no significant publication bias among articles included for each predictor variables. Overall assessment of publication bias from proportion of anaemia and bias among articles included for one predictor variable are described herewith as a sample. The Egger’s and Begg’s statistical tests are described for the rest of the predictor variables in the tables of the main document.

The overall bias was assessed by considering log of odds on horizontal axis and its standard error on vertical axis by considering r out of n sample were observed to have anaemia, leading to a proportion of r/n. The associated log odds is ln(r/(n-r)) with SE sqrt(1/r + 1/(n-r)). The plot indicates significant publication bias. It also further proved by egger’s test indicating, coefficient of bias is - 4.54 (95% CI: -7.94, -1.14), P = 0.010.

Figure 1: Funnel plot on assessment of overall publication bias for articles reporting proportion of anaemia

Assessment of publication bias among articles included in each of predicator variable indicates there is no significant bias. The funnel plot and statistical test (egger’s and begg’s test) were done for articles included for the different variables. From these, funnel plot and Egger test of articles included for one variable; birth interval, was indicate herewith as a sample.

Figure 2: Assessment of publication bias among the articles included to assess association of birth interval with anaemia.

**Egger's test for small-study effects:**

Regress standard normal deviate of intervention

Effect estimate against its standard error

Number of studies = 26 Root MSE = 2.216

-----------------------------------------------------------------------------------------------------

Std_Eff | Coef. Std. Err. t P>|t| [95% Conf. Interval]

-------------+--------------------------------------------------------------------------------------

slope | .2917074 .4008899 0.73 0.474 -.5356887 1.119103

bias | 1.123515 1.321756 0.85 0.404 -1.604455 3.851484

----------------------------------------------------------------------------------------------------

Test of H0: no small-study effects P = 0.404
